# Supplementary material for: Protection of the Ovine Fetal Gut against Ureaplasma-Induced Chorioamnionitis: A Potential Role for Plant Sterols
Source: Nutrients. 2019 Apr 27;11(5):968. doi: 10.3390/nu11050968 (PMC6566982; doi:10.3390/nu11050968)
Supplement: Supplementary file 1 [file nutrients-11-00968-s001.pdf]

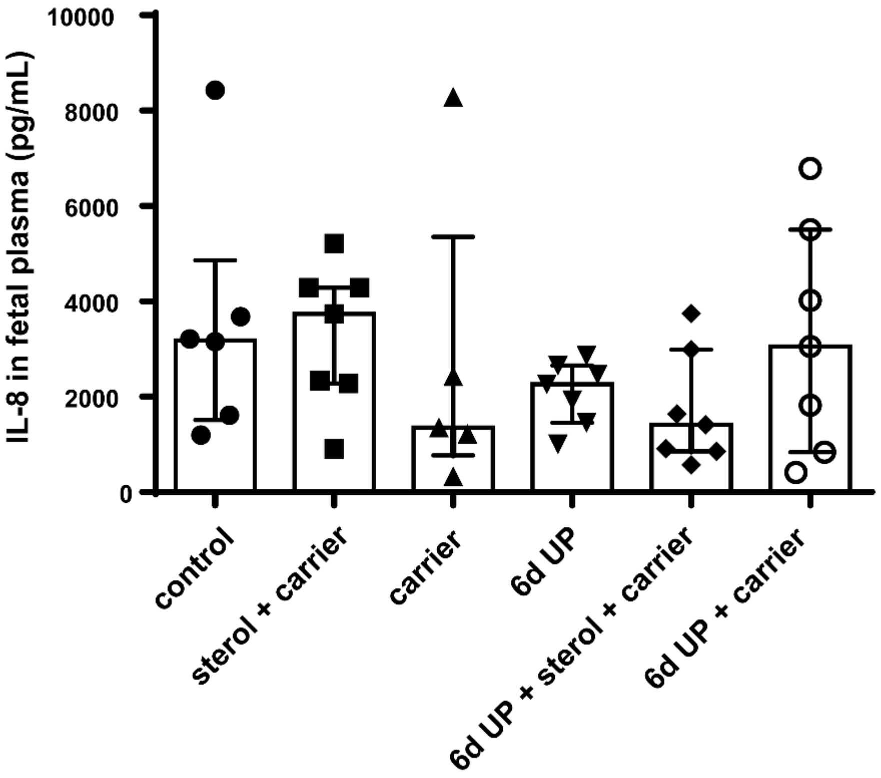

Supplemental figure 1. Circulatory IL-8 levels in fetuses of 133d GA. No significant changes were found between the treatment groups. UP: Ureaplasma

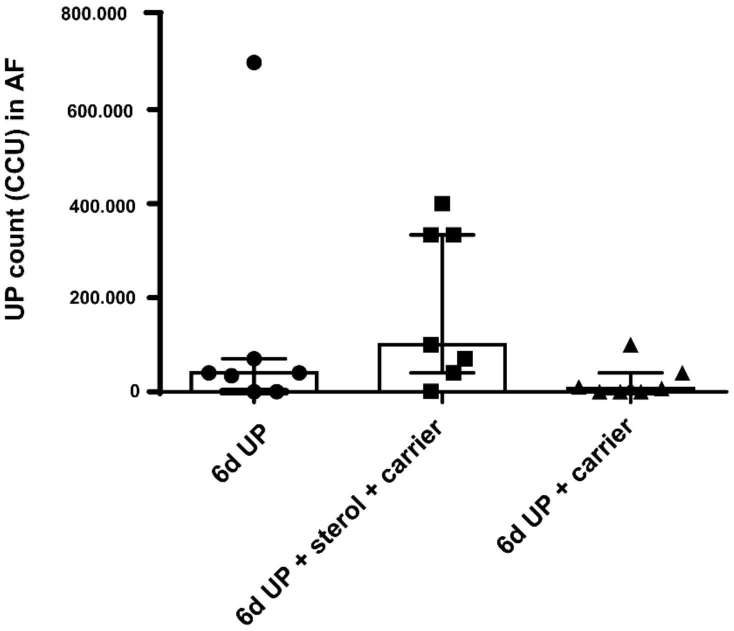

Supplemental figure 2. Ureaplasma parvum titers measured in AF samples taken at 133d GA. UP was only found in AF of animals injected with UP. No statistical differences were found between the different treatment groups. AF: amniotic fluid; CCU: color changing unit; GA: gestational age; UP: Ureaplasma.

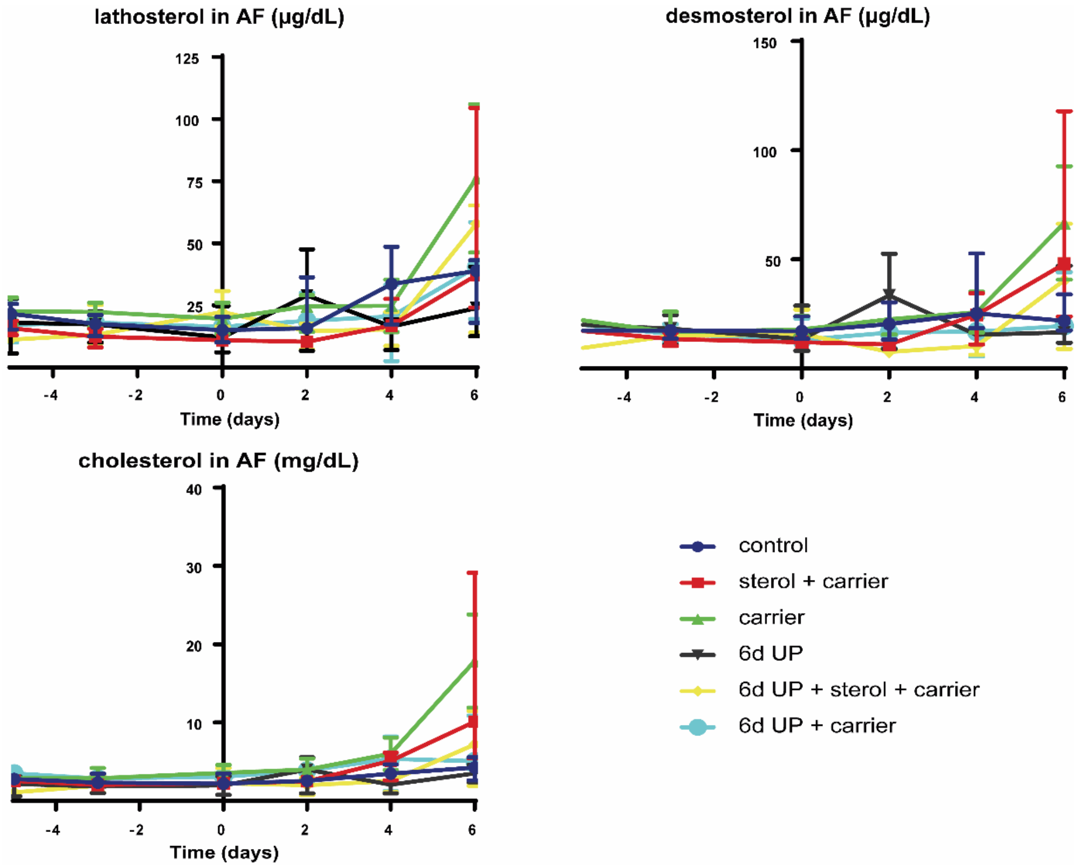

Supplemental figure 3. AF concentrations of cholesterol precursors (lathosterol, desmosterol) and cholesterol. At day 11, no significant differences were found for lathosterol (A), desmosterol (B) and cholesterol (C) in all treatment groups. Day 0 (122d GA) is the start of plant sterol treatment, day 5 (127d GA) is the day of intra-amniotic UP injection and day 11 (133d GA) is the moment of preterm delivery. AF: amniotic fluid; GA: gestational age; UP: Ureaplasma.

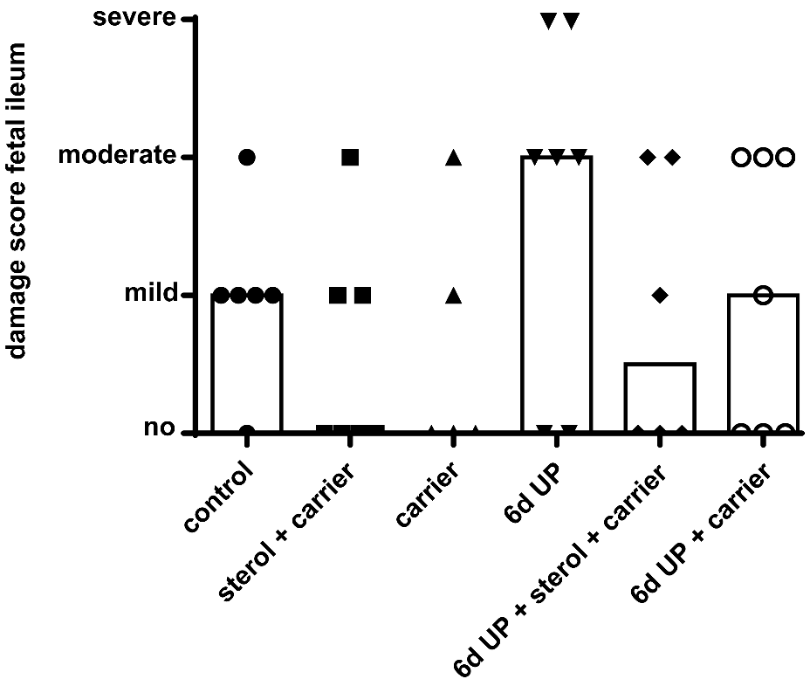

Supplemental figure 4. Evaluation of damage in the distal ileum of preterm lambs by H&E staining. Intestinal sections were judged as no damage (no damage visible), mild damage (apical epithelial integrity not complete, but no apparent loss of enterocytes), moderate damage (apical epithelial integrity not complete, loss of some enterocytes from the villus tips) or severe damage (apical epithelial integrity not complete, abundant loss of enterocytes from villus tips). The 6d UP group had more moderate to severe intestinal damage than the other experimental groups. H&E: hematoxylin and eosin; UP: Ureaplasma.

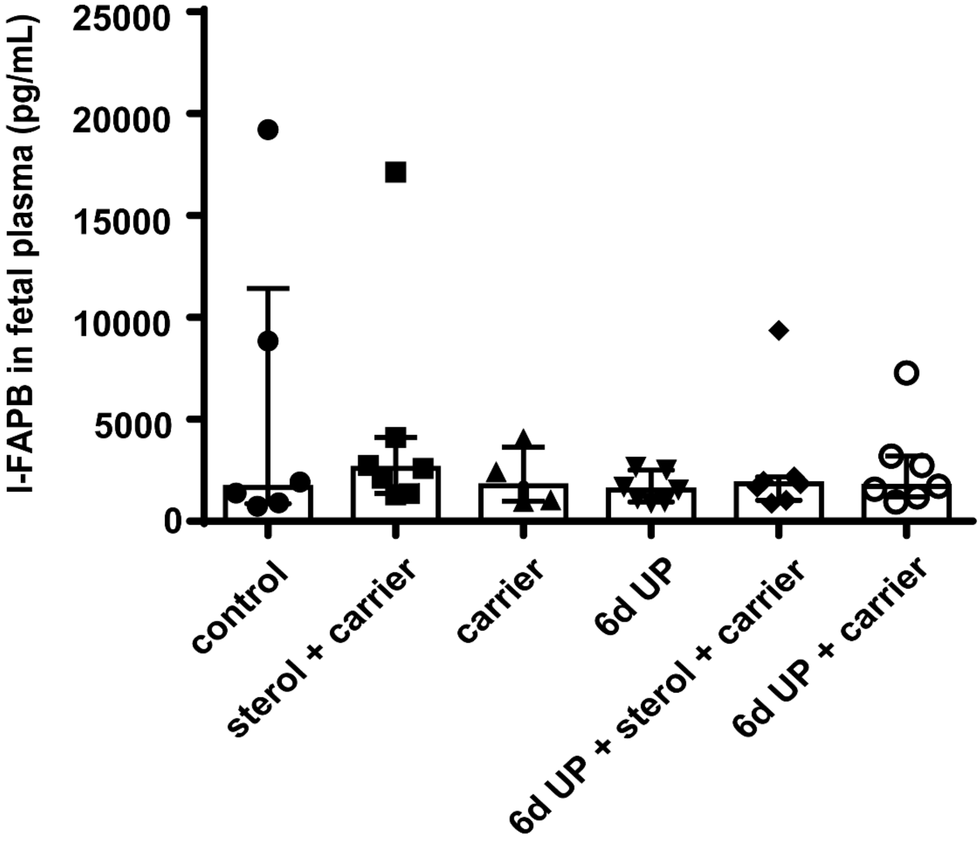

Supplemental figure 5. Circulatory I-FABP levels in fetuses of 133d GA. No significant changes were found between all treatment groups. I-FABP: intestinal fatty acid binding protein; UP: Ureaplasma.

Supplemental table 1. Identification of phosphocholines, sphingomyelins, phosphatidylinositol and bile acids.

Mass-to-charge ratio *m/z*	Exact mass	Δm (ppm)	Lipid assignment	Adduct	Fragment ions (*m/z*)
725.5587	725.5567	2.75	SM (34:1)	$[M+Na]^+$	184.0 (phosphocholine head group), 542.5 (loss of phosphocholine ion, Δm=183), 666.5 (loss of trimethylamine, Δm=59)
760.5868	760.5850	2.36	PC (34:1)	$[M+H]^+$	184.0 (phosphocholine head group), 577.5 (loss of phosphocholine ion, Δm=183), 701.5 (loss of trimethylamine, Δm=59)
782.5692	782.5670	2.8	PC (34:1)	$[M+Na]^+$	184.0 (phosphocholine head group), 577.5 (loss of phosphocholine ion + Na^+), 599.5 (loss of phosphocholine ion, Δm=183), , 723.5 (loss of trimethylamine, Δm=59)
810.6006	810.5983	2.83	PC (36:1)	$[M+Na]^+$	184.0 (phosphocholine head group), 605.5 (loss of phosphocholine ion + Na^+), 627.5 (loss of phosphocholine ion, Δm=183), 751.5 (loss of trimethylamine, Δm=59)
885.5423	885.5498	8.46	PI (38:4)	$[M-H]^-$	581.3 (loss of FA, Δm=304), 241,0 PI(241)
498.2901	498.2895	1.2	Taurodeoxycholic acid (TDCA)	$[M-H]^-$	79.96 $[SO_3]^-$, 106.9 $[C_2H_3O_3S]^-$, 124 $[C_2H_6NO_3S]^-$

514.2848	514.2844	0.7	Taurocholic acid (TCA)	$[M-H]^-$	79.96 $[SO_3]^-$, 106.9 $[C_2H_3O_3S]^-$, 124 $[C_2H_6NO_3S]^-$

Supplemental table 2. Identification of the carrier (2-hydroxypropyl-β-cyclodextrin).

	Mass-to-charge ratio *m/z* **on tissue**	Mass-to-charge ratio *m/z* **standard**	Δm (ppm)
Parent ion	1331.4856	1331.4854	0.15
MS/MS fragments	1169.4324	1169.4323	0.09
	1007.3794	1007.3794	0
	949.3375	949.3374	0.10
	845.3264	845.3266	0.23
	787.2846	787.2849	0.38
	729.2427	729.2428	0.14
	683.2736	683.2738	0.29
	625.2317	625.2318	0.16
	567.1898	567.1903	0.88
	463.1787	463.1787	0
	405.1369	405.1371	0.49
	304.2464	304.2434	9.86
